# Entomologic and molecular investigation into *Plasmodium vivax *transmission in Singapore, 2009

**DOI:** 10.1186/1475-2875-9-305

**Published:** 2010-10-29

**Authors:** Lee-Ching Ng, Kim-Sung Lee, Cheong-Huat Tan, Peng-Lim Ooi, Sai-Gek Lam-Phua, Raymond Lin, Sook-Cheng Pang, Yee-Ling Lai, Suhana Solhan, Pei-Pei Chan, Kit-Yin Wong, Swee-Tuan Ho, Indra Vythilingam

**Affiliations:** 1 Environmental Health Institute, National Environment Agency, 11 Biopolis Way Singapore 138667; 2 National Public Health Laboratory, Ministry of Health, College Road, Singapore 169854; 3 Ministry of Health, College Road, Singapore 169854; 4 Environmental Health Department, National Environment Agency. 40 Scotts Road, Singapore 228231

## Abstract

**Background:**

Singapore has been certified malaria free since November 1982 by the World Health Organization and despite occasional local transmission, the country has maintained the standing. In 2009, three clusters of malaria cases were reported in Singapore.

**Methods:**

Epidemiological, entomological and molecular studies were carried out to investigate the three clusters, namely Mandai-Sungei Kadut, Jurong Island and Sembawang.

**Results:**

A total of 29 malaria patients, with no recent travel history, were reported in the three clusters. Molecular analysis based on the *msp3α *and *msp1 *genes showed two independent local transmissions: one in Mandai-Sungei Kadut and another in Sembawang. Almost all cases within each cluster were epidemiologically linked. In Jurong Island cluster, epidemiological link remains uncertain, as almost all cases had a unique genetic profile. Only two cases shared a common profile and were found to be linked to the Mandai-Sungei Kadut cluster. Entomological investigation found *Anopheles sinensis *to be the predominant Anopheline in the two areas where local transmission of *P. vivax *was confirmed. *Anopheles sinensis *was found to be attracted to human bait and bites as early as 19:45 hrs. However, all *Anopheles *mosquitoes caught were negative for sporozoites and oocysts by dissection.

**Conclusion:**

Investigation of *P. vivax *cases from the three cluster areas confirmed the occurrence of local transmission in two areas. Although *An. sinensis *was the predominant Anopheline found in areas with confirmed transmission, the vector/s responsible for the outbreaks still remains cryptic.

## Background

Singapore has been certified malaria free since November 1982 by the World Health Organization [1] and despite occasional local transmission, the country has maintained the standing due to its comprehensive system that prevents the re-establishment of malaria viz. vector surveillance and control, early case detection, and aggressive preventive and remedial actions upon detection of cases [2,3]. The incidence of reported malaria declined substantially from 8.0 per 100,000 population in 1977 to 2.0 per 100,000 population in 2007. In recent years, incidence has been maintained at between 2.0 and 2.6 per 100,000 population. Most infections were caused by *Plasmodium vivax *(66-78%), and *Plasmodium falciparum *(19-31%) [3]. Some 91-98% of all reported cases were imported, with 90% of them originating from neighbouring endemic countries in Southeast Asia and from the Indian subcontinent. These imported cases included foreign workers on employment pass, local residents who contracted the disease abroad, foreigners seeking medical treatment in Singapore, and foreign tourists [3].

Singapore's vulnerability to malaria is accentuated by its status as a trade and travel hub, high dependency on foreign workers from neighboring endemic countries, and increased regional travel to and from malarious areas. It has been reported that 60% of vivax malaria cases occur in Southeast Asia and the Western Pacific, and that India contributes substantially to this regional disease burden [4]. The presence of pockets of *Anopheles *vectors further render Singapore receptive to malaria. Through studies conducted in the 1960s, malaria vectors of Singapore were established as *Anopheles epiroticus (sundaicus) *and *Anopheles maculatus *[5]. *Anopheles letifer *has also been suspected to be a vector [6].

To reduce the threat of outbreaks, the National Environment Agency has identified specific malaria receptive areas for regular *Anopheles *surveillance and control [2]. Between 1983 and 2007, a total of 29 outbreaks involving 196 local cases were reported. These outbreaks had median size of three cases. Half (14) of these outbreaks were identified on the main island, while the rest occurred on off-shore islands. Either *An. sundaicus *or *An. maculatus *were found in 13 outbreaks, while no vectors could be determined in the others [3]. In 2006, 13 vivax malaria cases involving foreign workers from endemic countries were reported at the Jurong island [7]. Larvae surveillance and adult trapping did not yield any *Anopheles *mosquitoes.

For vivax malaria, difficulties exist in differentiating relapsed cases from local transmission. In May-Aug 2009, 29 cases of vivax malaria were again detected at three different cluster locations in Singapore. This report highlights the use of entomologic and molecular techniques to assist in epidemiologic investigations.

## Methods

### Human blood samples and molecular confirmation of diagnosis

**Figure 1:**
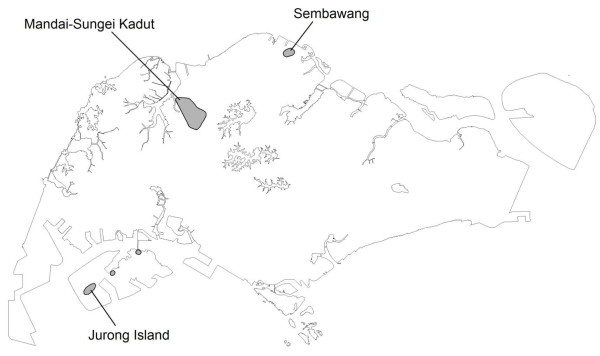
**Spatial map of the three malaria clusters, which were located at the peripheral of the island city**.

Between May and July 2009, a total of 29 vivax malaria cases were identified as working/living in three separate cluster locations in Singapore with no overseas travel history of note. They comprised 16 cases at Mandai-Sungei Kadut (1°24'23.15"N, 103°45'28.88"E), 9 cases at Jurong Island (1°15'42.49"N, 103°40'37.68"E), and 4 cases at Sembawang (1°27'10.42"N, 103°49'58.13"E) (Figure 1).

A total of 20 residual blood samples from the three cluster locations (7 from Jurong Island, 10 from Mandai-Sungei Kadut, and 3 from Sembawang) were obtained from various hospitals for sequencing and phylogenetic analysis. As part of the national public health programme, DNA was extracted from whole blood using QIAamp DNA blood kit (QIAGEN, USA), following the manufacturer's recommendations, and subjected to molecular analysis. Nested PCR assay for detection of malaria parasites was carried out as previously described [8,9]. In addition to testing the four human malaria parasite species, the samples were also tested for *Plasmodium knowlesi*.

### Molecular analysis of malarial parasites

#### Restriction fragment length polymorphism of *msp3α *of *P. vivax*

The polymorphic region of *msp3α *gene of *P. vivax *was amplified by PCR as previously described [10]. PCR amplification for each sample was carried out in 50 μl reaction mixture containing 1× reaction buffer, 2 mM MgCl_2_, 200 mM of each deoxynucleoside triphosphate, 0.25 μM each of primers, 1.25 U of Taq DNA polymerase (Promega, USA) and 5 μl of extracted genomic DNA was used for each reaction. PCR cycling were carried out using the Veriti™ thermal cycler (Applied Biosystem, CA, USA) using the following parameters: initial denaturation at 94°C for 10 min, 35 cycles at 94°C for 1 min, 56°C for 1 min and 72°C for 2 min 30s, followed by a final extension step of 68°C for 10 min. Five μl of PCR product of msp3*α *gene was digested individually with restriction enzyme *Alu*I and *Hha*I (Promega, USA) in 20 μl reactions and incubated at 37°C for 4 hr. The digested DNA fragments were then analysed by agarose gel electrophoresis.

#### Cloning, sequencing and phylogenetic analysis of *msp1 *gene of the parasite

The *msp1 *gene of *P. vivax *was PCR amplified as described previously [11]. The PCR amplified *msp1 *gene fragment was cloned into TOPO Zero Blunt vector (Invitrogen, USA). Ten to thirty clones were sequenced with M13 primers. Each of the *msp1 *gene sequence obtained was compared with those from the Genbank database using Basic Local Alignment Search Tool (BLAST). Sequences were aligned using ClustalX [12] and phylogenetic analysis was performed using MEGA4 programme [13]. Phylogenetic tree was constructed using the neighbour-joining method based on Kimura 2-parameter distance matrix including transitions and transversions. Reliability of the phylogenetic tree was accessed using the bootstrap methods with 1000 replicates.

#### Adult mosquito collection

Larval surveillance was performed by the public health officers from the National Environment Agency's regional offices. Adult mosquito collections, by the same field officers and laboratory based officers from the Environmental Health Institute (EHI), were performed from 7 pm to 1 am. Collections were done using bare-leg catch method [14] and the human baited net trap [15]. All mosquitoes were brought into the EHI laboratory for identification and examination. Entomological investigation at Mandai-Sungei Kadut was performed from 30 May to 26 August, which was initiated about two weeks after the onset date of the first case and ended four weeks after the final reported case. At Jurong island, the surveillance was performed from 26 May to 2 September, while onset of reported cases were from 3 May to 26 July. The surveillance at Sembawang was from 29 July to 1 September, in response to cases with onset dates from 12 June to 12 July.

### Mosquito identification and examination

#### Morphological identification

Taxonomic keys of Harrison and Scanlon; Reid [16,17] were used for the morphological identification. *Anopheles *mosquitoes were dissected to extract ovaries to determine parity and the midguts and salivary glands were examined for oocysts and sporozoites respectively.

#### Molecular identification - sequencing of internal transcribed spacer 2 (ITS2) region

All mosquitoes that could not be identified accurately, due to missing pertinent taxonomic features were subjected to molecular and phylogenetic analysis. DNA was extracted from the legs of the *Anopheles *mosquitoes using the DNeasy tissue Kit (Qiagen USA) following the manufacturer's recommendations. The rDNA ITS2 was amplified using a previously described protocol [18]. PCR products were analysed by agarose gel electrophoresis stained with ethidium bromide. PCR products were purified using Purelink™ PCR Purification kit (Invitrogen, USA). Sequencing was done by a commercial laboratory according to the BigDye Terminator Cycle Sequencing kit (Applied Biosystems) protocol.

Consensus sequence from each mosquito was obtained by assembling a contiguous sequence from raw sequencing data using Seqman software (Lasergene, DNASTAR, USA). Sequences obtained from each mosquito were aligned with ClustalW using Megalign software (Lasergene, DNASTAR, USA). The ITS2 gene sequences obtained from this study were phylogenetically compared to other members of the *Anopheles hyrcanus *group in GenBank. The phylogenetic trees were constructed using the method described for molecular analysis of malarial parasite.

## Results

### Epidemiologic findings

Using nested PCR assay, *P. vivax *was confirmed to be the only malaria parasite present in all 20 samples sent to EHI for further analysis. The Mandai-Sungei Kadut cluster comprised 14 foreign workers and two local residents who developed onset of symptoms between 16 May and 1 July 2009. Mandai and Sungei Kadut are two adjacent industrial estates located in the northern part of Singapore adjacent to a tropical rainforest. At the time of the outbreak, there were ongoing earthworks for the construction of a subway line. The foreign workers were from four different countries viz. India, Thailand, Bangladesh and Malaysia.

The Jurong Island cluster comprised nine foreign workers who developed onset of symptoms between 3 May and 26 July 2009. Jurong Island, located southwest of the mainland, is a 32 km^2 ^island that houses major petrochemical industries. The cases, all men aged 24-46 years, were from India and Bangladesh but none of them could furnish a history of past malaria infection. Epidemiologic investigations traced an earlier imported case (designated as J4), who was ill on 4 April 2009 and had previous malaria in India in Jun 2008 to be the likely source of infection for the local cases.

The Sembawang cluster comprised three local residents and one foreign worker (an Indian national) who developed onset of symptoms between 12 June and 12 July 2009. Sembawang is a semi-rural location in the north of Singapore that is lined by shophouses and grass nurseries adjacent to a tropical rainforest.

### Molecular epidemiology - analysis of *P. vivax msp3α *and *msp1 *genes

**Figure 2:**
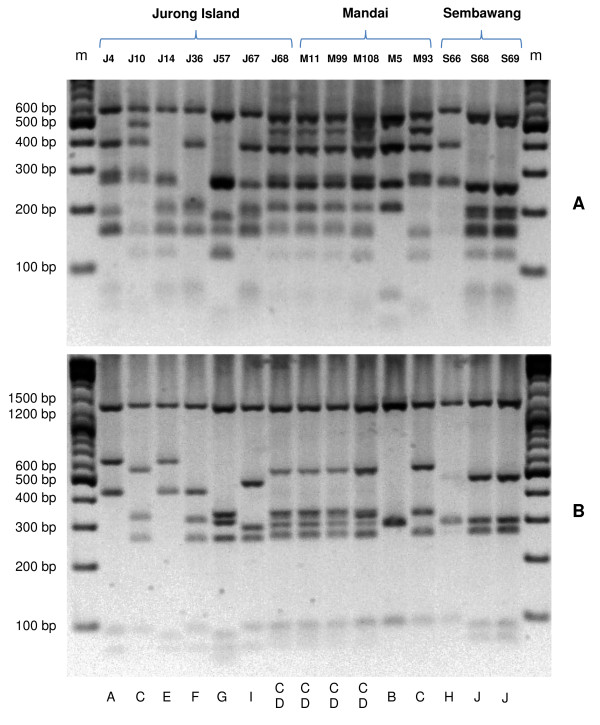
**Profile of restriction fragment length polymorphism (RFLP) of *P. vivax msp3α *gene**. Restriction enzymes digestion using *Alu*I (A) and *Hha*I (B) enzymes for samples from Jurong Island (J), Mandai Estate (M) and Sembawang (S). The profile of J68, M11, M99 and M108 were similar to other 5 isolates from Mandai Estate (data not shown). DNA size markers in base-pairs (bp) are shown in the end lanes (m). Alphabets at the bottom of the gel represent specific genotypes, deciphered by eyeballing, present in each sample. Sample with mixed genotype infections is indicated with more than one alphabet.

The PCR-RFLP analysis of the *msp3α *gene demonstrated that *P. vivax *parasites in samples from Jurong Island, Mandai-Sungei Kadut and Sembawang were highly diverse. Ten unique patterns of the PCR-RFLP profile were observed among the 20 samples analyzed (Figure 2). Moreover, the sum of RFLP fragments size derived for most Mandai-Sungei Kadut cases (M9, M11, M108, M84, M71, M99, M82 and M80) and a Jurong Island case (J68) was greater than the size of corresponding undigested PCR product, indicating the presence of multiple haplotypes of *msp3α *in each of these samples. Each of the plausible haplotypes was visually elucidated from the RFLP pattern and assigned an alphabet: A-J and the possible *msp*3*α *haplotypes for each case were scored in Table 1.

**Table 1:** Comparison between analysis by RFLP of *msp3α *gene and sequencing of *msp1 *gene of *P. vivax*.

Location	Sample	*msp3α *RFLP pattern	*msp1 *haplotype
Jurong Island	SG(EHI)-J10	C	Hap1, Hap2
	SG(EHI)-J14	E	Hap5, Hap6
	SG(EHI)-J36	F	Hap3, Hap4
	SG(EHI)-J57	G	Hap14
	SG(EHI)-J67	I	Hap18
	SG(EHI)-J68	C, D	Hap1, Hap2
			
Mandai Estate	SG(EHI)-M5	B	Hap30
	SG(EHI)-M9	C, D	Hap1, Hap2
	SG(EHI)-M11	C, D	Hap1, Hap2, Hap15
	SG(EHI)-M71	C, D	Hap2, Hap13, Hap16
	SG(EHI)-M80	C, D	Hap1, Hap2, Hap12
	SG(EHI)-M82	C, D	Hap1, Hap2
	SG(EHI)-M84	C, D	Hap1, Hap2
	SG(EHI)-M93	C	Hap1, Hap2, Hap9, Hap11
	SG(EHI)-M99	C, D	Hap1, Hap2, Hap17
	SG(EHI)-M108	C, D	Hap1, Hap2, Hap7, Hap8
			
Sembawang	SG(EHI)-S66	H	Hap19, Hap22, Hap23, Hap24, Hap25, Hap28, Hap29
	SG(EHI)-S68	J	Hap10, Hap20, Hap21, Hap26, Hap27
	SG(EHI)-S69	J	Hap10

Each alphabet for *msp3α *RFLP pattern represents a unique genotype based on analysis of fragment size.

**Figure 3:**
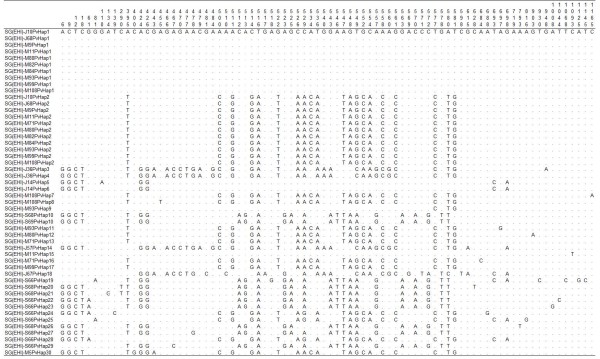
**Sequence polymorphisms in the gene encoding the C-terminal 42 kDa fragment of *msp1 *of *P. vivax *in samples from Singapore (SG(EHI))**. Haplotypes derived from specific locations are prefixed with specific alphabet; J = Jurong Island, M = Mandai Estate and S = Sembawang. Identical sequences are prefixed with identical haplotype (Hap) number. Nucleotide positions are numbered vertically above polymorphic sites. Dots represent identical nucleotide residues and dashes represent deletions. DNA sequences were deposited in Genbank under the accession numbers GU971656 to GU971705.

The analysis of the gene encoding the C-terminal 42 kDa fragment (1,230 bp) of *P. vivax msp1 *in 17 cases from Jurong Island (n = 4), Mandai-Sungei Kadut (n = 10) and Sembawang (n = 3) further confirmed the results derived from the RFLP of *msp3α *gene. An average of 7 clones were sequenced from each case (Additional file 1). Based on the *msp1 *gene sequences, all except 4 samples had multiple genotypes (2-5 genotypes per individual) of *P. vivax*. As expected, cloning and sequencing of the *msp1 *gene provide a higher resolution analysis as multiple *msp1 *haplotypes were also identified in samples detected as having single *P. vivax *genotype by RFLP method (Table 1). Sequence alignment revealed 30 haplotypes of *msp1 *gene, characterized by specific point mutations (Figure 3). Additional file 1 provides details on the haplotype of each clone sequenced.

### Mandai-Sungei Kadut cluster

Analysis of 10 samples taken from Mandai-Sungei Kadut showed that eight samples, including two local Singaporean cases (M11 and M99) had an identical PCR-RFLP pattern, which was generated by at least 2 haplotypes (C and D) (Table 1). For the other two samples, M93 pattern appeared to comprise C only, while M5 was completely different (as revealed by the *HhaI *digest). The suspected index case, M5, was thus molecularly found to be unrelated to the cluster. The *msp3α *PCR-RFLP finding was supported by *msp1 *sequencing, which revealed that all Mandai-Sungei Kadut cases, except M5 shared haplotypes 1 and/or 2 (Table 1). This molecular data strongly supports the epidemiological finding of local transmission of *P. vivax *malaria at Mandai-Sungei Kadut, and suggested that M5 was not the index case of the outbreak and was not linked to the outbreak.

### Jurong Island cluster

Analysis of seven samples from Jurong Island revealed seven unique PCR-RFLP patterns (Figure 2). Analysis of the *msp1 *sequences of six cases also revealed distinct haplotypes among the cases (Table 1). Interestingly, two cases (J10 and J68) had the C and CD RFLP pattern of *msp3α*, that were characteristic of Mandai-Sungei Kadut cases. The possible link between these two cases with the Mandai-Sungei Kadut outbreak was again substantiated by the *msp1 *sequences, where haplotypes 1 and 2 were found in the two cases (J10 and J68) (Table 1).

### Sembawang cluster

Analysis of samples from Sembawang revealed two unique RFLP patterns (*msp*3*α*) that were distinct from those sampled in Jurong Island and Mandai-Sungei Kadut. Two samples (S68 and S69), which belonged to local Singaporeans had identical RFLP pattern, while the foreigner (S66) had a different haplotype. Based on this analysis, it is highly possible that local transmission of *P. vivax *was taking place in Sembawang but none of the cases were associated with the malaria clusters in Jurong Island and Mandai-Sungei Kadut. Case S66, though living in the area, was not linked to the cluster in Sembawang. The results from *msp1 *sequence analysis were consistent with that of *msp3α *RFLP. Haplotype 10 was shared between the two local residents (S68 and S69), while S66 had no common haplotype with any of the two.

### Phylogenetic analysis of parasite genes

**Figure 4:**
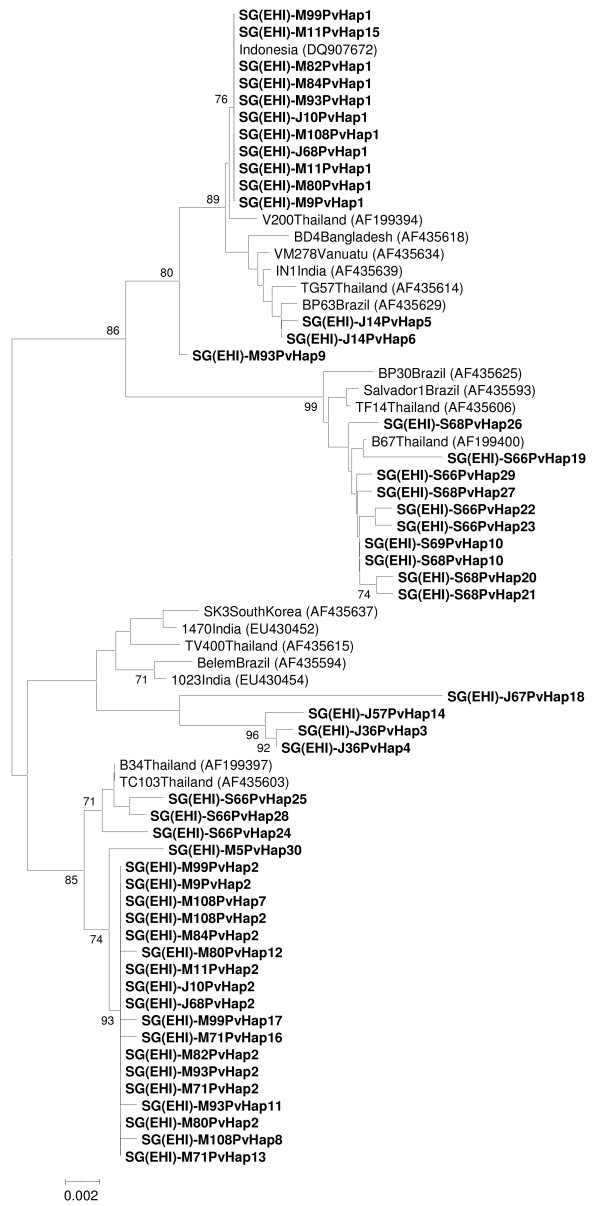
**Neighbour-joining tree of *P. vivax *based on the gene encoding the C-terminal 42 kDa fragment of *msp1***. Samples from Singapore are in bold and prefixed according to location: J = Jurong Island, M = Mandai Estate, S = Sembawang. Figures on the branches are bootstrap percentages based on 1,000 replicates and only those above 70% are shown. GenBank accession numbers are in brackets.

Phylogenetic analysis based on the *msp1 *gene showed that *P. vivax *parasites from these three malaria clusters can be subdivided into three distinct clades, each with a strong bootstrap support (Figure 4). Two most common haplotypes (haplotypes 1 and 2) that were shared among samples from Mandai-Sungei Kadut and two of Jurong Island cases fell into two separate clades (I and III). Haplotype 1 (clade I) was most closely related to *P. vivax *from Indonesia, whereas the closest relatives of haplotype 2 (clade III) were those previously reported in Thailand. For samples from Jurong Island, most haplotypes branched out independently and clustered with *P. vivax *strains from various countries such as India, Brazil and Thailand. This further supports the diversity of lineages among the samples from Jurong Island. Although multiple haplotypes of *msp1 *of *P. vivax *were found in samples from Sembawang, they were notably distinct from those found in Jurong Island and Mandai-Sungei Kadut. The Sembawang *P. vivax msp1 *haplotypes formed a tight clade (clade II) and grouped with the Thai and Brazilian strains of *P. vivax*. Though the source of the parasite cannot be concluded from the molecular study, the results suggest that the *P. vi*vax within these three malaria clusters were derived from parasite lineages that were commonly circulating within this region.

### Entomological investigation

**Figure 5:**
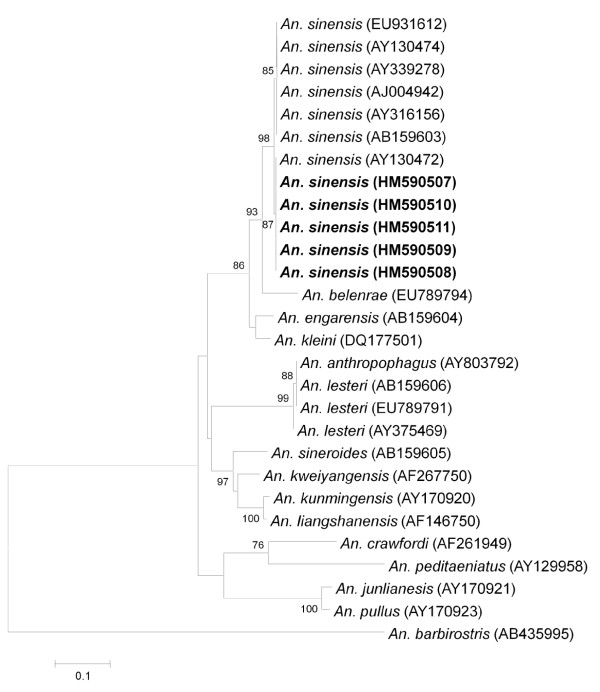
**Phylogenetic tree based on the gene encoding the internal transcribed spacer region 2 (ITS2) of *Anopheles hyrcanus *Group produced by the neighbour-joining method**. Those highlighted in bold are sequences obtained from Singapore mosquitoes and clustered in *An. sinensis *clade. Figures on the branches are bootstrap percentages based on 1000 replicates and only those above 70% are shown. *Anopheles barbirostris *were used as outgroup. GenBank accession numbers are in brackets.

All adult mosquitoes caught from the malaria cluster area and larval samples collected from various breeding places were identified morphologically (Additional File 2). The predominant anopheline mosquitoes caught in Mandai-Sungei Kadut and Sembawang were *An. sinensis*. Due to the wide morphological variations observed in *An. sinensis *and the presence of several species belonging to the *An. hyrcanus *group in Singapore, all adult mosquitoes caught belonging to this species group that have the pertinent taxonomic characteristic missing (e.g. scales fallen off), were subjected to molecular analysis and were all confirmed to be *An. sinensis *(Figure 5). Phylogenetic analysis inferred from the NJ method showed that the ITS2 genes of adult *An. sinensis *caught at the cluster areas formed a monophyletic clade with other *An. sinensis *caught in other parts of Singapore and those in Japan, South Korea, China and Thailand. Percent divergent of *An. sinensis *caught in Singapore ranges from 0.5 to 1.3% when compared to the same species caught elsewhere.

In Jurong Island, adult mosquito surveillance yielded only *Culex *species and no *Anopheles*; and larval surveillance detected *Aedomyia *species, *Culex *species and *An. separatus *within 1 km around the living quarter of the cases. However, one breeding with two larvae of *An. sinensis *was detected in a drain in a vacant land more than 1 km away from the living quarters.

In Mandai-Sungei Kadut and Sembawang, a total of 98 adult *Anopheles *mosquitoes were obtained from the cluster areas as shown in Additional File 2. In Mandai-Sungei Kadut, *An. sinensis *was the predominant anopheline mosquito (n = 67) and one *An. karwari *was also caught. Larvae surveillance around the residence and workplace of cases that spread across 3 km, yielded four breedings of *An. sinensis *and one of a member of the *An. barbirostris *complex. *Anopheles sinensis *was found in drains, ground puddle, and disused quarry filled with water; and *An. barbirostris *was found in ground puddle.

In Sembawang, *An. sinensis *was the only adult Anopheline caught (n = 30). Larval surveillance also found 17 *An. sinensis *breedings in earth drains and ground puddles, within 1 km from the implicated location of transmission. No other Anopheline larvae were found.

Man biting/seeking *An. sinensis *was caught along the forest fringe. Interestingly, in Mandai-Sungei Kadut, they were also caught biting men beside well-lit busy major asphalt road. In Sembawang, they were caught in dimly lit open-air restaurants. They were found to bite humans as early as 19:45 hour and were active till midnight. All anophelines were negative for oocyst and sporozoites by dissection.

### Parous rate, life expectancy and vectorial capacity of *Anopheles sinensis*

The efficiency of a mosquito as a vector depends on how long it lives and how frequently it feeds on humans. The gonotrophic cycle of *An. sinensis *is three days [19]. The parous rate of *An. sinensis *was 26.9 and 34.8 in Mandai-Sungei Kadut and Sembawang respectively as shown in Table 2. The life expectancy of *An. sinensis *was determined using the formula of Garrett-Jones and Grab [20]. The vectorial capacity of *An. sinensis *using the formula of Garrett-Jones and Shidrawi [21] was 0.04 and 0.17 for Mandai-Sungei Kadut and Sembawang respectively as shown in Table 2. From these results, it is evident that only 1.3 and 2.8% of the mosquitoes would be expected to live the 10 days necessary for the sporozoites to be formed. Those surviving the 10 days would have a further life expectancy of less than 1 day.

**Table 2:** Parous rate, life expectancy and vectorial capacity of *Anopheles sinensis*

	Mandai-Sungei Kadut	Sembawang
Total Number	67	30

Bites/Man/Night	4.2	2.2

Parous rate	26.9	34.8

Probability of survival (p)	0.65	0.7

1/-log_e_p (life expectancy)	2.32	2.80

p^10^(%)	1.3	2.82

p^10^/-log_e_p (days)	0.03	0.08

Vectorial capacity	0.04	0.17

p^10^: percentage of population expected to live long enough to become infective with an extrinsic cycle of 10 days; p^10^/-log_e_p: subsequent life expectancy

Though the most direct measurable index of transmission is the entomological inoculation rate, the rate cannot be determined, as these surveys yielded no infected mosquito.

## Discussion

Recent records and this report show that the inherent risk of malaria transmission in Singapore demands continuous vigilance [2,3,22,23]. However, with the possibility of relapse of vivax malaria cases among the large population of foreign workers from endemic countries, determining if a cluster is due to local transmission can be challenging. The hypnozoite stage of *P. vivax *can be dormant in infected liver cells for months or years and relapse of vivax cases are well known and documented. A study in India has shown that the northern *P. vivax *population was highly polymorphic and a high relapse rate of 40% has been observed [24]. In Singapore, some recent local transmissions, such as the incidents in Jurong Island in 2006 and 2009 involved Indians and Bangladeshis only, and hence, despite the absence of any past medical history of malaria infection, the contribution of relapsed cases to these clusters remained uncertain.

The use of molecular techniques was instrumental in confirming two of the local clusters (Mandai-Sungei Kadut and Sembawang) to be outbreaks. Among the Mandai-Sungei Kadut local cases, RFLP analysis of the *msp3α *gene revealed that all, except one, shared the same RFLP profile, which is generated by at least two genotypes. Further cloning and sequencing of the *msp1 *gene confirmed the epidemiological link among all the cases, and the multiple genotypes carried by each individual case. The same situation was found in Sembawang. However, both the *msp3α *RFLP and *msp1 *sequence analysis of Jurong Island cases showed that only two cases were molecularly linked, and they were also linked to the Mandai-Sungei Kadut outbreak.

In contrast to the outbreaks at Mandai-Sungei Kadut and Sembawang, events at Jurong Island presented a more interesting enigma. There were nine cases that tested positive for *P. vivax *and detailed inquiry revealed no recent travel history. However, molecular epidemiology revealed unique PCR-RFLP patterns among the cases and vector findings showed only evidence of *Anopheles separatus*, a non-vector, as the only Anopheline found within 1 km from the cases. A similar incident occurred in 2006 in the same area, when 13 cases were found among foreign workers from malaria endemic regions, and no *Anopheles *was found in that location. The molecular and entomological data suggest a possible coincident relapse among the cases at Jurong Island, and data are in agreement with epidemiologic data, which showed that only foreign workers from endemic countries were involved in the Jurong island cluster. Alternatively, this may reflect the limit of the molecular techniques in finding common haplotypes when only a single blood specimen was obtained from each worker. The dynamics of disease transmission at Jurong Island are still not fully understood and clearly warrant further entomologic and molecular studies.

Interestingly, where there was substantial molecular evidence of local transmission, adult *An. sinensis *was predominant, and multiple breedings of the Anopheline were found around the vicinity. There are no reports of *An. sinensis *being a vector in Southeast Asia. In the Malayan region, including Singapore, *An. sinensis *has not been considered a vector, since it was found to be more zoophagic and no malaria parasite sporozoites have been found in the mosquito [16,25]. In Thailand, *An. sinensis *has been considered only as a refractory vector with weak transmission capability [16,26]. However, *An. sinensis *has been incriminated as a vector of malaria in Northeast Asia viz. Korea and China [27-32]. In a recent experiment carried out on the susceptibility of different strains of *An. sinensis *towards Korean and Thai strains *P. vivax*, it was found that *An. sinensis *from Korea was able to develop oocysts and sporozoites against local strain of *P. vivax *and those from Thailand. On the other hand, when fed with *P. vivax *(Thailand strain), *An. sinensis *from Thailand failed to develop sporozoites [33]. This clearly demonstrated the possible variation in terms of the susceptibility of the different geographical strains of *An. sinensis *to malaria parasites. Park and co-workers reported that the sequences of ITS2 and COII among 10 Asian *An. sinensis *strains from China, Japan, Korea and Thailand were almost identical to one another with very small sequence variation (< 1.5% in both regions), despite its relatively wide geographical distribution range of > 2000 mi [34]. In this current study, the local strains of *An. sinensis *has also been found to be very similar (genetic divergent 0.5 to 1.3%) to the single cosmopolitan species concluded by Park and coworkers. Despite the varying vectorial status of the species demonstrated by laboratory experiment and field epidemiological evidences, current molecular techniques are not able to discriminate between vector strain and non-vector strain. Genetic features that better represent vector status should be of research interest.

In the two recent Singapore outbreaks in Mandai and Sembawang, *An. sinensis *has been the predominant species and its apparent intrusion into the urban setting that fringes the rainforest may suggest some level of adaptation. Furthermore, its biting period appeared to coincide with the period (7:45 pm to 12 am) of human activity. Whether *An. sinensis *is a malaria vector in Singapore, facilitated by the adaptation, remains elusive and the vector of the two small concurrent outbreaks remains cryptic.

Studies in Korea have shown that human biting rate of *An. sinensis *has been 87.5 bites/man/night and the vectorial capacity was 0.081 [19]. In our brief study, though the vectorial capacity was similar in Singapore (0.04 and 0.17), the bites/man/night was much lower, below 5. It thus seems that the high transmission potential may be due to the high human biting rate of the mosquito.

Results from this investigation strongly suggests that *An. sinensis*, which has never been implicated as a vector in Singapore, was the predominant Anopheline in Mandai-Sungei Kadut and Sembawang. In these areas, there was substantial molecular evidence of local transmission and multiple breedings of the Anopheline were found. Currently, work is ongoing to determine the vectorial status of *An. sinensis*. The human blood index is not known and no sporozoites have been found. The implication of *An. sinensis *as a vector would be significant, as this may result in the need to redefine boundaries of malaria receptive areas which are presently based on the geographical distributions in Singapore of *An. maculatus *and *An. epiroticus *(= *An. sundaicus*) [5].

Since it is critical to detect early any local transmission for control measures to be effective, the precautionary principle is applied by the public health authorities such that every clustering of cases in place and time is deemed to be a local outbreak until proven otherwise. Molecular tools and techniques can elucidate the parasite genotype or strain and the data can be used to track and link cases in an outbreak, but these techniques take time to be developed and validated. Meanwhile, interviews to determine case movements and previous infection remain the mainstay in outbreak prevention and control. Moving forward, this study shows how entomologic and molecular tools can be applied in epidemiologic investigation to provide an understanding of outbreaks. If conducted promptly and regularly, the data can be useful for assessment and mitigation of transmission.

## Competing interests

The authors declare that they have no competing interests.

## Authors' contributions

NLC, IV and LKS conceived the study and were responsible for the preparation of the manuscript. RL, SS and KT were responsible for diagnosis and epidemiological investigation. NLC, IV, LKS, PSG, TCH, PSC, HO were responsible field collection, supervision, identification and processing of mosquitoes. LKS and LYL conducted the molecular studies of all cases. TCH and PSC conducted molecular studies on the vectors. All authors have read and approved the manuscript.

## Supplementary Material

Additional file 1 **Number of clones of each *msp1 *haplotype derived from each *P. vivax *case**. Click here for file

Additional file 2 **Mosquito species obtained from the malaria outbreak areas**. Click here for file
