# Supplementary material for: Entomologic and molecular investigation into Plasmodium vivax transmission in Singapore, 2009
Source: Malar J. 2010 Oct 29;9:305. doi: 10.1186/1475-2875-9-305 (PMC2988040; doi:10.1186/1475-2875-9-305)
Supplement: Additional file 1 — Number of clones of each msp1 haplotype derived from each P. vivax case. [file 1475-2875-9-305-S1.PDF]

Additional file 1

Number of clones of each *msp1* haplotype derived from each *P. vivax* case

Location	Sample	Total no. of clones	Number of clones																													
			Hap 1	Hap 2	Hap 3	Hap 4	Hap 5	Hap 6	Hap 7	Hap 8	Hap 9	Hap 10	Hap 11	Hap 12	Hap 13	Hap 14	Hap 15	Hap 16	Hap 17	Hap 18	Hap 19	Hap 20	Hap 21	Hap 22	Hap 23	Hap 24	Hap 25	Hap 26	Hap 27	Hap 28	Hap 29	Hap 30
Jurong	J10	8	1	4	-	-	-	-	-	-	-	-	-	-	-	-	-	-	-	-	-	-	-	-	-	-	-	-	-	-	-	-
Jurong	J14	18	-	-	-	-	1	4	-	-	-	-	-	-	-	-	-	-	-	-	-	-	-	-	-	-	-	-	-	-	-	-
Jurong	J36	30	-	-	6	2	-	-	-	-	-	-	-	-	-	-	-	-	-	-	-	-	-	-	-	-	-	-	-	-	-	-
Jurong	J57	33	-	-	-	-	-	-	-	-	-	-	-	-	-	10	-	-	-	-	-	-	-	-	-	-	-	-	-	-	-	-
Jurong	J67	8	-	-	-	-	-	-	-	-	-	-	-	-	-	-	-	-	-	1	-	-	-	-	-	-	-	-	-	-	-	-
Jurong	J68	7	4	3	-	-	-	-	-	-	-	-	-	-	-	-	-	-	-	-	-	-	-	-	-	-	-	-	-	-	-	-
Mandai	M5	10	-	-	-	-	-	-	-	-	-	-	-	-	-	-	-	-	-	-	-	-	-	-	-	-	-	-	-	-	-	7
Mandai	M9	4	1	3	-	-	-	-	-	-	-	-	-	-	-	-	-	-	-	-	-	-	-	-	-	-	-	-	-	-	-	-
Mandai	M11	28	3	2	-	-	-	-	-	-	-	-	-	-	-	-	3	-	-	-	-	-	-	-	-	-	-	-	-	-	-	-
Mandai	M71	10	0	6	-	-	-	-	-	-	-	-	-	-	1	-	-	1	-	-	-	-	-	-	-	-	-	-	-	-	-	-
Mandai	M80	10	5	4	-	-	-	-	-	-	-	-	-	1	-	-	-		-	-	-	-	-	-	-	-	-	-	-	-	-	-
Mandai	M82	10	2	8	-	-	-	-	-	-	-	-	-	-	-	-	-	-	-	-	-	-	-	-	-	-	-	-	-	-	-	-
Mandai	M84	10	2	5	-	-	-	-	-	-	-	-	-	-	-	-	-	-	-	-	-	-	-	-	-	-	-	-	-	-	-	-
Mandai	M93	10	6	2	-	-	-	-	-	-	1		1	-	-	-	-	-	-	-	-	-	-	-	-	-	-	-	-	-	-	-
Mandai	M99	10	7	1	-	-	-	-	-	-	-	-	-	-	-	-	-	-	1	-	-	-	-	-	-	-	-	-	-	-	-	-
Mandai	M108	13	1	5	-	-	-	-	1	1	-	-	-	-	-	-	-	-	-	-	-	-	-	-	-	-	-	-	-	-	-	-
Semb	S66	7	-	-	-	-	-	-	-	-	-	-	-	-	-	-	-	-	-	-	1	-	-	1	1	1	1	-	-	1	1	-
Semb	S68	7	-	-	-	-	-	-	-	-	-	3	-	-	-	-	-	-	-	-	-	1	1	-	-	-	-	1	1	-	-	-
Semb	S69	9	-	-	-	-	-	-	-	-	-	1	-	-	-	-	-	-	-	-	-	-	-	-	-	-	-	-	-	-	-	-

Semb: Sembawang
