# Supplementary material for: Entomologic and molecular investigation into Plasmodium vivax transmission in Singapore, 2009
Source: Malar J. 2010 Oct 29;9:305. doi: 10.1186/1475-2875-9-305 (PMC2988040; doi:10.1186/1475-2875-9-305)
Supplement: Additional file 2 — Mosquito species obtained from the malaria outbreak areas. [file 1475-2875-9-305-S2.PDF]

Additional File 2

Mosquito species obtained in the malaria outbreak areas

	Mandai Estate		Sembawang		Jurong Island	
Mosquito species	Adults	Larvae	Adults	Larvae	Adults	Larvae
Anopheles barbirostris		2				
An. karwari	1					
An. separatus						2
An. sinensis	67	4	30	16		2
Aedes albopictus			56	310		
Ae. butleri				10		
Ae. longirostris				2		
Aedes sps	4		20	50		
Aedomyia sp						21
Coquillettidia crassipes			1			
Coquillettidia sp			1			
Culex annulus				15		
Cx. brevipalpis				10		
Cx. fuscanus				31	30	110
Cx. gelidus			2			
Cx. nigropunctatus			2	25		
Cx. pseudovishnui				21		
Cx.quinquefasciatus			1	50		
Cx. rubithoracis				2		
Cx. sitiens				2		
Cx. spathifurca				5		
Cx. tritaeniorhynchus			7			
Cx. vishnui			30			
Cx. vishnui sp complex			8			
Cx. sp	19		267			
